# The effect of hemostatic dressing prototypes for the uniformed services on selected blood coagulation parameters in pigs

**DOI:** 10.1186/s13028-017-0297-9

**Published:** 2017-05-12

**Authors:** Zbigniew Adamiak, Wioletta Krystkiewicz, Andrzej Pomianowski, Danuta Bukowiecka, Waldemar Zubrzycki, Marek Jałyński, Piotr Holak, Joanna Głodek, Paweł Jastrzębski

**Affiliations:** 1 Department of Surgery and Radiology, Faculty of Veterinary Medicine, University of Warmia and Mazury in Olsztyn, Oczapowskiego 14, 10-957 Olsztyn, Poland; 2 Department of Internal Medicine, Faculty of Veterinary Medicine, University of Warmia and Mazury in Olsztyn, Oczapowskiego 14, 10-957 Olsztyn, Poland; 3 Police Academy in Szczytno, Marszałka Józefa Piłsudskiego 111, 12-100 Szczytno, Poland

**Keywords:** Hemorrhage, Hemostatic dressing, Pigs

## Abstract

**Background:**

Serious injuries accompanied by severe bleeding are life-threatening. Post-traumatic hemorrhage involves the risk of developing coagulopathy. Hemostatic dressings are widely used to minimize bleeding. The application of procoagulants in control of hemorrhage may lead to thrombosis or disseminated intravascular coagulation. The aim of this study was to evaluate the effect of hemostatic dressing prototypes on the porcine coagulation system.

**Results:**

Fibrinogen and d-dimer concentrations were significantly higher in the experimental groups where hemostatic dressings were used in comparison with the control group. Considerable differences in antithrombin III activity and thrombin–antithrombin complex concentrations were also observed between groups.

**Conclusions:**

The hemostatic dressing comprising modified seton impregnated with 18.0 g/m^2^ of procoagulant was most effective in preserving the physiological equilibrium between fibrinogenesis and fibrinolysis.

## Background

Serious injuries accompanied by severe bleeding are life-threatening and pose a significant challenge for physicians and veterinarians participating in rescue actions. In an emergency setting, damaged vessels are often treated with hemostatic dressings, which are left in the wound site until surgical repair [1]. Global rescue standards, including Trauma Life Support and Tactical Combat Causality Care, include detailed guidelines for using hemostatic dressings.

Hemostatic dressings are widely available on the market and they are entering into mainstream use [2]. They differ in the form of application (powder, sponge, cloth), active ingredient which stimulates the clotting system, time to effective hemostasis and side effects [3]. Post-traumatic increases the risk of coagulopathy in patients who have never been diagnosed with clotting disorders.

Hemostasis is an integrated process, which leads to the attainment of an equilibrium between various factors that inhibit and activate blood clotting. In serious injuries, control of hemorrhage with the use of procoagulants can lead to thrombosis and disseminated intravascular coagulation (DIC) [4]. The choice of the type and amount of procoagulants that induce hemostasis without causing thrombosis or DIC is not an easy task.

The aim of this study was to evaluate the effect of selected prototypes of hemostatic dressings on selected blood parameters in pigs.

## Methods

The experiment was performed on 30 pigs divided into five groups. Prototypes of hemostatic dressings were tested in three groups of animals. Animals were premedicated with atropine (Atropinum Sulfuricum, Polfa-Warszawa, Warsaw, Poland) at 0.05 mg/kg body weight intramuscular (BW IM), and azaperone (Stresnil, Janssen Pharmaceutica N.V. Tumhoutseweg, Beerse, Belgium) at 2.5 mg/kg BW IM. General anesthesia was induced with ketamine (Bioketan, Vetoquinol, Gorzów Wielkopolski, Poland) at 8 mg/kg BW IM and maintained with propofol (Scanofol, Vetoquinol, Gorzów Wielkopolski, Poland) at 10 mg/kg body weight intravenous (BW IV). Tracheal intubation with normoventilation was performed. The surgery area in the region of the left inguinal fossa was prepared, and a lateral incision was made across the femoral artery. A selected hemostatic dressing was applied to the wound. Group I animals were treated with the OBR/G/S sponge (dressing material sponge made of Na–Ca chitosan/algal composite microfibers and nanofibers), group II pigs—with OBR/MBT/S (tactic gauze modified with a polymer mixture of Na–Ca chitosan/algal composite microfibers and nanofibers) impregnated with medium levels of procoagulants (22.9 g/m^2^), and group III animals—with OBR/MS/S (seton gauze modified with a polymer mixture of Na–Ca chitosan/algal composite microfibers and nanofibers) impregnated with medium levels of procoagulants (18.0 g/m^2^). The coagulants in all dressings were chitosan (ChitoClear hqg 95) and sodium alginate (Protanal LF10/60 FT). Hemostatic dressings were sterilized by electron beam irradiation. They were used to control bleeding from surgically incised femoral arteries in pigs. The animals were transported to the post-operative recovery room after bleeding had stopped. Group IV animals were treated with non-hemostatic dressing. In all group IV pigs, the applied dressing did not stop bleeding, and the animals died 1–12 h after surgery. The control group consisted of healthy animals whose femoral arteries were not incised and which were not treated with the hemostatic dressings. Blood samples were collected from control group pigs for comparative analyses.

Blood for hematology was collected into test tubes coated with EDTAK_2_ (ethylenediaminetetraacetic acid dipotassium salt dehydrate), and blood for coagulation tests was collected into test tubes containing 3.2% sodium citrate. Blood was sampled from each animal in five replications to evaluate changes in coagulation and fibrinolytic systems: before the surgical procedure, 1 h after the procedure, 12 h after the procedure, 24 h after the procedure, and 7 days after the procedure.

The following parameters were determined in hematology tests: white blood cell counts (WBC), red blood cell counts (RBC), hematocrit (Hct), hemoglobin concentrations (Hgb), mean corpuscular volume (MCV), mean corpuscular hemoglobin (MCH), mean corpuscular hemoglobin concentrations (MCHC), platelet counts (PLT) and mean platelet volume (MPV).

The following parameters were determined in coagulation tests: activated partial thromboplastin time (APTT), prothrombin time (PT), thrombin time (TT), fibrinogen concentrations (FIB), d-dimer concentrations (DD), antithrombin III activity (ATIII) and thrombin–antithrombin complex concentrations (TAT).

Data were expressed as the mean (±SD) for six animals per group. Statistical analysis was performed using one-way analysis of variance (ANOVA) followed by the Bonferroni post hoc test. SigmaPlot Software Version 12.0 (Systat Software Inc., San Jose, USA) was used for statistical analysis. The power analysis was performed using G*Power Software version 3.1.9.2.

## Results

### Hematology tests

The mean  values of hematological parameters for each group are presented on the Table 1. Total WBC counts did not change significantly 1 h after surgery, but 12 h after the procedure, WBC counts doubled in groups II and III relative to control (statistically significant difference), and in group II, the same difference relative to control was noted before surgery. In group II, the difference in WBC counts decreased 24 h after the procedure, but remained statistically significant.

Minor but significant differences in RBC counts were observed before the surgery relative to control. In group II, the remaining parameters—HCT and MCV—differed significantly between 12 and 24 h after the procedure.

Platelet counts decreased in all groups 1–12 h after surgery. Platelet counts began to increase 24 h after the procedure, and this trend was maintained until the end of the first week after surgery. In blood samples collected before the procedure, significant differences were noted in group III relative to groups I and IV and the control group. Those differences were maintained in samples collected 12 h, 24 h and 1 week after surgery. One hour after the procedure, MPV decreased in groups I–III on, whereas a minor increase in MPV was noted in the control group and group IV. In groups I–III, MPV began to increase 12 h after surgery. Significant differences in MPV values were observed between groups I–III and the control group. Differences were deemed significant when the P values were <0.05. For presented results the power of the statistical tests using for hematology parameters estimated 0.71–0.95.

**Table 1:** Mean values of hematological parameters

Group	Time of sampling	WBC	RBC	HGB	HCT	MCV	MCH	MCHC	PLT	MPV
		10^3^/µl	10^6^/µl	g/dl	%	fl	pg	g/dl	10^3^/µl	fl
I	0′	22.23	6.56	10.18	35.34	54.08	15.54	28.78	507.00	7.56
	1 h	23.71	5.78	9.08	30.37	52.80	15.67	29.63	500.50	6.88
	12 h	22.08	5.78	9.17	30.95	53.72	15.93	29.68	513.33	6.93
	24 h	21.15	5.43	8.68	28.85	53.33	16.05	30.10	559.67	6.93
	1 week	15.28	5.72	10.23	30.87	54.13	17.95	33.13	610.33	7.25
II	0′	32.51	5.915	9.82	31.68	52.96	16.38	30.96	636.4	6.62
	1 h	27.63	6.11	9.96	32.12	52.60	16.32	30.98	579.80	6.44
	12 h	30.87	6.29	10.28	33.50	53.28	16.36	30.70	609.80	6.86
	24 h	35.44	6.28	10.12	33.12	52.80	16.16	30.62	563.00	6.72
	1 week	22.01	5.69	9.30	29.88	52.63	16.38	31.13	782.50	6.20
III	0′	26.60	6.18	9.90	32.56	52.69	15.99	30.37	656.57	6.89
	1 h	21.83	5.81	9.24	30.23	52.03	15.91	30.60	564.86	6.53
	12 h	27.24	5.99	9.62	31.36	52.30	16.03	30.67	602.68	6.67
	24 h	34.33	5.71	9.09	29.67	51.90	15.86	30.57	586.00	6.37
	1 week	21.53	5.87	9.30	30.53	51.94	15.83	30.44	722.43	7.00
IV	0′	16.76	6.15	9.98	32.56	54.8	16.63	31.15	395.0	8.06
	1 h	18.77	5.78	9.48	30.97	55.85	15.63	29.35	365.5	8.67
Control	0′	17.96	5.83	11.03	33.07	56.77	18.93	33.47	366.33	8.37
	12 h	15.66	5.03	9.13	28.60	57.03	18.20	31.87	370.67	8.47
	24 h	21.6	5.08	9.325	28.025	55.6	18.425	33.175	339.5	8.025

### Coagulation tests

The mean  values of coagulation parameters for each group are presented on the Table 2. Prothrombin time did not change significantly within groups on successive sampling dates. In all experimental groups, PT values were significantly shorter than in the control group.

**Table 2:** Mean values of coagulation parameters

Group	Time of sampling	APTT	TT	PT	FIB	DD	AT III	TAT
		S	S	S	g/l	µg/l	%	ng/ml
I	0′	30.46	22.62	13.06	4.64	290.6	109.4	39
	1 h	25.96	22.8	13.92	5.672	538.2	104.8	44.2
	12 h	21.35	14.55	13.92	10.76	462.83	90.83	128.17
	24 h	62.95	14.38	14.62	11.33	631.33	85.00	185.33
	1 week	65.10	19.78	14.18	6.94	369.67	102.00	76.33
II	0′	33.8	23.62	10.8	11.256	462.6	115.8	27.2
	1 h	37.96	20.08	10.30	11.95	689.40	114.20	30.20
	12 h	30.64	17.84	11.66	10.90	439.80	95.20	114.20
	24 h	31.82	22.80	10.58	10.94	888.20	88.60	113.20
	1 week	28.45	26.03	11.25	10.40	1002.50	99.50	85.75
III	0′	26.24	21.93	11.56	9.33	437.43	116.00	26.29
	1 h	27.60	19.47	11.01	10.12	278.71	109.43	47.14
	12 h	29.33	18.94	12.04	10.45	425.43	89.86	193.14
	24 h	30.21	27.31	10.97	10.68	358.29	80.00	196.29
	1 week	23.03	25.04	15.78	10.86	838.58	95.57	104.29
IV	0′	29.57	21.83	11.21	4.55	366.75	113.16	30.66
	1 h	29.75	19.06	10.6	9.48	493.25	95.33	44.33
Control	0′	35.05	24.48	15.78	4.07	376.25	108.50	31.00
	12 h	33.90	25.46	16.70	4.53	462.83	101.0	40.25
	24 h	36.23	22.85	18.05	4.26	393.25	113.00	50.50

Activated partial thromboplastin time remained fairly constant, but it increased threefold in group I 24 h after the procedure relative to the values determined 12 h after the surgery, which resulted in significant differences between group I and the remaining groups.

Thrombin time was significantly shortened 12 h after the procedure. The greatest decrease in TT was noted 12 h after surgery, and significant differences in TT values were noted between groups I–III and the control group.

Significant differences were also observed in the values of the remaining coagulation parameters: FIB, DD, ATIII and TAT.

Fibrinogen concentrations increased in all experimental groups 1, 12 and 24 h after the procedure. Fibrinogen concentrations in the experimental groups were twofold higher than in the control group 1, 12 and 24 h after surgery. One week after the procedure, FIB values decreased significantly in all experimental groups, which minimized the significant differences between groups I and III vs. the control group. Due to a minor drop in FIB values in group II 1 week after the procedure, the difference between group II and the control group remained statistically significant.

d-Dimer concentrations were significantly higher in all experimental groups relative to control. One hour after the procedure, DD values increased in groups I, II and IV, but decreased below baseline values in group III. d-Dimer concentrations decreased in groups I and III and increased in group II 12 h after the surgery. An increase in DD values in groups I and II, and a minor decrease in the value of this parameter in group III were observed 24 h after the procedure. One week after surgery, DD values decreased nearly twofold in group I, whereas a continued increase in this parameter was noted in groups II and III.

The activity of ATIII decreased significantly 12 h after surgery and remained low in blood samples analyzed 24 h after the procedure. The concentration of TAT increased significantly at the corresponding times. Significant differences in ATIII and TAT values were noted 24 h after surgery relative to control. The difference in TAT concentration was highly significant, and the analyzed parameter was approximately threefold higher in the experimental groups than in the control group.

Differences were deemed significant when the P values were <0.05. For presented results the power of the statistical tests using for coagulation parameters were >0.9.

## Discussion

The main role of hemostasis is to maintain the fluidity of circulating blood, preserve the integrity of the intravascular compartment and prevent blood loss when endothelial continuity of a blood vessel is broken. The pathophysiology of hemostasis describes the interactions between coagulation, anticoagulation and fibrinolysis. Thrombin is the main enzyme responsible for blood clotting. It converts soluble fibrinogen to insoluble fibrin via extrinsic and intrinsic pathways [5].

Disseminated intravascular coagulation (DIC) is caused by the widespread activation of the clotting cascade and the generation of fibrin. Fibrin binds blood platelets and leads to the formation of blood clots. The clotting process uses large amounts of blood platelets and fibrinogen, which leads to DIC [6]. In DIC, the function of coagulation inhibitors, in particular antithrombin (AT) and protein C, is impaired, which leads to excessive production of thrombin [7].

High fibrinogen levels in all experimental groups, relative to the control group, could point to the activation of coagulation processes. An increase in fibrinogen concentrations can lead to hypercoagulation and, if accompanied by other factors such as factor XII (Hageman factor), to DIC [8].

High d-dimer concentrations are correlated with the levels of selected inflammatory cytokines [9], which suggests that similarly to fibrinogen, elevated d-dimer values contribute to thrombosis and inflammatory processes [10]. The most significant changes in the concentrations of d-dimers and fibrinogen were noted in group II where the application of procoagulants with hemostatic dressings could lead to thrombosis, DIC or acute inflammation. The highest d-dimer values were noted 1 week after the procedure. Similar results were noted after surgery by Chojnowski [11].

Significant changes were observed in ATIII activity and TAT concentrations. The application of procoagulants which enhance the physiological effects of thrombin activated intrinsic inhibitors, mainly ATIII [12]. Antithrombin III deactivates thrombin by forming biologically inactive TAT complexes with an estimated half-life of 15 min [13]. The strongest correlation between a decrease in ATIII activity and an increase in TAT concentrations indicates that the applied procoagulants were most effective in group III animals. In every experimental group, high TAT levels were observed 12–24 h after the surgical procedure. In view of the short half-life of TAT, the results of this experiment indicate that thrombin was most effectively inhibited in group III.

Platelet counts were considerably higher in all experimental animals than in control pigs, and they increased in all experimental groups 1 week after surgery. An increase in platelet counts was not observed in previous samplings. Thrombocytes release adrenalin, noradrenalin, serotonin and thromboxane to promote vasoconstriction and minimize bleeding after injury. Thrombocytes aggregate to form platelet plugs, which stop bleeding. The absence of a clear increase in platelet counts immediately after injury could point to the effectiveness of both procoagulants in the tested hemostatic dressings. The increase in thrombocyte counts 1 week after the procedure suggests their involvement in intravascular coagulation processes. High thrombocyte counts could contribute to both venous and arterial thrombosis.

## Conclusions

The results of this study indicate that the physiological equilibrium between fibrinogenesis and fibrinolysis was most effectively preserved in group III animals-with OBR/MS/S modified seton impregnated with medium levels of procoagulants (18.0 g/m^2^) Modified seton impregnated with medium levels of the procoagulant (18.0 g/m^2^) can be effectively used to develop a new, effective and safe hemostatic dressing for controlling severe bleeding from large arteries.

## Abbreviations

APTT: activated partial thromboplastin time
ATIII: antithrombin III activity
ChitoClear hqg 95: initial chitosan
DD: d-dimer concentrations
DIC: disseminated intravascular coagulation
EDTAK_2_: ethylenediaminetetraacetic acid dipotassium salt dihydrate
FIB: fibrinogen concentrations
Hct: hematocrit
Hgb: hemoglobin concentrations
MCH: mean corpuscular hemoglobin
MCHC: mean corpuscular hemoglobin concentrations
MCV: mean corpuscular volume
MPV: mean platelet volume
OBR/G/S: dressing material sponge made of Na–Ca chitosan/algal composite microfibers and nanofibers
OBR/MBT: tactic gauze modified with a polymer mixture of Na–Ca chitosan/algal composite microfibers and nanofibers
OBR/MS/S: seton gauze modified with a polymer mixture of Na–Ca chitosan/algal composite microfibers and nanofibers
PLT: platelet counts
Protanal LF10/60/FT: initial alginate
PT: prothrombin time
RBC: red blood cell counts
TAT: thrombin–antithrombin complex concentrations
TT: thrombin time
WBC: white blood cell counts
